# The understanding of congruent and incongruent referential gaze in 17-month-old infants: an eye-tracking study comparing human and robot

**DOI:** 10.1038/s41598-020-69140-6

**Published:** 2020-07-17

**Authors:** F. Manzi, M. Ishikawa, C. Di Dio, S. Itakura, T. Kanda, H. Ishiguro, D. Massaro, A. Marchetti

**Affiliations:** 1 Research Unit on Theory of Mind, Department of Psychology, Università Cattolica del Sacro Cuore, Milan, Italy; 2 School of Graduated Letter, Department of Psychology, Kyoto University, Kyoto, Japan; 3 Centre for Baby Science, Doshisha University, Kyoto, Japan; 4 Human-Robot Interaction Laboratory, Department of Computer Science, Kyoto University, Kyoto, Japan; 5 Advanced Telecommunications Research Institute International, IRC/HIL, Keihanna Science City, Kyoto, Japan; 6 Department of Systems Innovation, Osaka University, Toyonaka, Japan

**Keywords:** Neuroscience, Psychology

## Abstract

Several studies have shown that the human gaze, but not the robot gaze, has significant effects on infant social cognition and facilitate social engagement. The present study investigates early understanding of the referential nature of gaze by comparing—through the eye-tracking technique—infants’ response to human and robot’s gaze. Data were acquired on thirty-two 17-month-old infants, watching four video clips, where either a human or a humanoid robot performed an action on a target. The agent’s gaze was either turned to the target (congruent) or opposite to it (incongruent). The results generally showed that, independent of the agent, the infants attended longer at the face area compared to the hand and target. Additionally, the effect of referential gaze on infants’ attention to the target was greater when infants watched the human compared to the robot’s action. These results suggest the presence, in infants, of two distinct levels of gaze-following mechanisms: one recognizing the other as a potential interactive partner, the second recognizing partner's agency. In this study, infants recognized the robot as a potential interactive partner, whereas ascribed agency more readily to the human, thus suggesting that the process of generalizability of gazing behaviour to non-humans is not immediate.

## Introduction

Human infants display a sensitivity towards the human eyes from birth, a phenomenon known as preference for direct gaze^1^. Direct gaze has been suggested to activate social brain networks and facilitate social cognition in infants^2^. Additionally, some studies have shown that averted gaze also affects infants’ cognitive processing of objects and faces^3,4^. It has been shown that new-born babies can discriminate between direct and averted gaze as they are faster to make saccades to peripheral targets cued by direct gaze^5^. Such sensitivity to other’s eye-gaze may be regarded as precursor of gaze following in later development, which is essential for efficient social learning^6^. In experimental settings, gaze following has been studied in infants as young as 3 months^7^. Data generally show that infants begin to follow eye-gaze at about 6 months^8–10^. Infants have also shown to be able to use information provided by eye-gaze to understand intentions behind actions. For example, it has been shown that infants use other’s gaze direction to predict or anticipate action^11^. In the study by Phillips and colleagues^11^, an actor grasped one of two objects in a situation where cues from the actor's gaze could serve to determine which object would be grasped. The results showed that 12- and 14-month-olds, but not 8-month-olds, recognized that the actor was likely to grasp the object which she looked at before grasping, in line with the referential nature of the actor’s gaze.

In the present study, we explored early understanding of referential gaze toward a target when infants observed either a human or a humanoid robot performing an action toward an object in a gaze-following task^12^. This task resembles the classical visuo-spatial cueing paradigm^13^ used to evaluate the process of attention shift, i.e. visuo-spatial orienting^14–16^, toward the same direction/object/event that another person is attending to. Using a gaze-following task, Senju et al.^17^ studied the referential gaze with infants. The authors recorded ERPs of adults and 9-month-old infants while watching scenes containing a human congruent-object gaze shifts or incongruent-object gaze shifts. The ERP results suggest that 9-month-olds process the object-congruent gaze shifts similarly to adults. In addition, an early frontal component was observed in infants, which showed greater amplitude in response to congruent gaze. This component may reflect a rapid processing of socially relevant information, such as the identification of communicative or informative situations, and could provide a basis for the development of attention sharing, social learning and Theory of Mind (ToM).

With respect to robots, several studies have shown that people tend to perceive robots as interactive partners^18^, although—with increasing age—generally attribute to them poor human-like mental qualities^19–21^. Only a few studies, however, have investigated early socio-cognitive responses to robots’ behaviour^22,23^. While the human gaze has been shown to have significant effects on infants’ social cognition and facilitate social engagement^4,24^, social cognitive studies with robots have failed to find an effect of the robot gaze on infants’ behaviour. For example, while reporting that 12-months-old infants do follow the robot gaze direction, Okumura et al.^22^ also found that the robot gaze does not facilitate the learning processes of a new object. Nevertheless, infants appear to become sensible to the direction of the robot gaze if they first observe the robot engaging in social interaction with an adult^25^. Likewise, infants imitate the robot’s goal-directed actions only when the robot first establishes eye contact with an adult^26^. These findings suggest that the robot gaze may not be as meaningful to the infant as the human gaze, although it can be charged of social meaning through a triadic social engagement, where the human adult provides a role model of the relationship.

Additionally, it has also been shown that infants do not display anticipatory gaze in response to robots’ action, suggesting a lack of intentionality attribution to the robot^23^. In support of this observation, Kanakogi and Itakura^27^ found that 10-month-old infants showed anticipatory looking at human reaching actions, but no anticipation in response to a mechanical hand. Furthermore, a brain imaging study using functional near-infrared spectroscopy (fNIRS) examined infants’ brain activation while watching either functional or non-functional actions performed by a human and a mechanical hand^28^. The result showed that the left middle-posterior temporal cortex responded selectively to the human hand, but only in the context of functionally relevant actions on objects. This evidences infants’ sensitivity to agency when a meaningful action is performed by a human and not by robots.

Altogether, these findings seem to suggest poor involvement of early social cognition when facing a robotic agent. Also, there is no direct evidence of infants’ sensitivity to the robot’s action when paired with the robot referential gaze, which represents an extremely important cue enhancing social engagement. To this purpose, we monitored the gazing behaviour of 17-month-olds infants while watching video-clips showing either a human or a humanoid robot performing an action. We selectively involved 17-month-old infants because at this age infants are able to follow the gaze of others^29^ and have reached an understanding of the referential nature of the gaze^7,30–34^. The action performed by the agents was either congruently or incongruently anticipated by the direction of the agent’s gaze. Additionally, to enhance socio-cognitive engagement, we also had the human and robot agents either performing or not direct eye-contact to the infant before action performance. Based on previous findings, we hypothesized to find the following: (1) infants would be equally sensitive to the effect of direct eye-contact on attention by both agents; (2) infants would be more sensitive to the referential gaze of both agents in the congruent with respect to the incongruent condition; (3) in the congruent condition, infants would be more sensitive to the referential gaze of the human with respect to the robot agent.

## Results

### Total fixation duration

**Figure 1:**
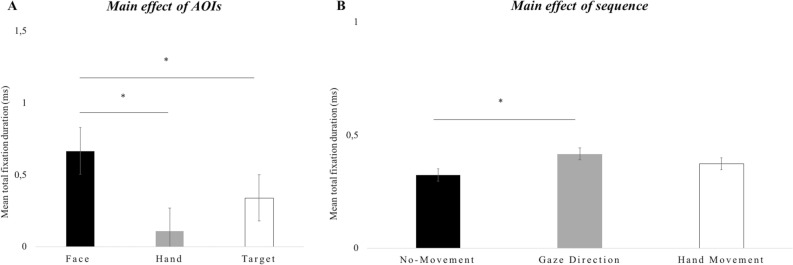
(**A**–**B**). Infants’ total fixation duration scores. (**A**) The graph shows the mean scores (ms) for each area of interest (Face, Hand, Target) across eye-contact, gaze direction, video sequence and agent; (**B**) The graph shows the mean scores (ms) for each video sequence (No-Movement, Gaze-Direction, Hand-Movement) across areas eye-contact, gaze direction, area of interest and agent. The bars represent the standard error of the mean. * indicates significant differences.

The results showed a main effect of area of interests (*AOIs*), *F*(2, 27) = 50.628, *p* < 0.001, *partial-η*^*2*^ = 0.789, *δ* = 1, indicating that, independent of eye-contact, gaze direction, AOIs and agency, infants exhibited a greater fixation time on the face compared to both the hand, *M*diff = 0.557, *SE* = 0.05, *p* < 0.001 and the target, *M*diff = 0.326, *SE* = 0.07, *p* < 0.001. Additionally, infants’ general attention was greater to the target compare to the hand, *M*diff = 0.231, *SE* = 0.048, *p* < 0.001 (Fig. 1A). Additionally, the results revealed a main effect of *sequence*, *F*(2, 56) = 5.187, *p* < 0.01, *partial-η*^*2*^ = 0.15, *δ* = 0.80, indicating that, independent of eye-contact, gaze direction, AOIs and agency, infants exhibited a shorter total fixation time in sequence 2 (Gaze-Direction), compared to sequence 1 (No-Movement), *M*diff = − 0.093, *SE* = 0.25, *p* < 0.01, but not with respect to sequence 3 (Hand-Movement). This main effect is plotted in Fig. 1B. Overall, both results highlight the relevance, for infants, of the face region and gazing behaviour.

**Figure 2:**
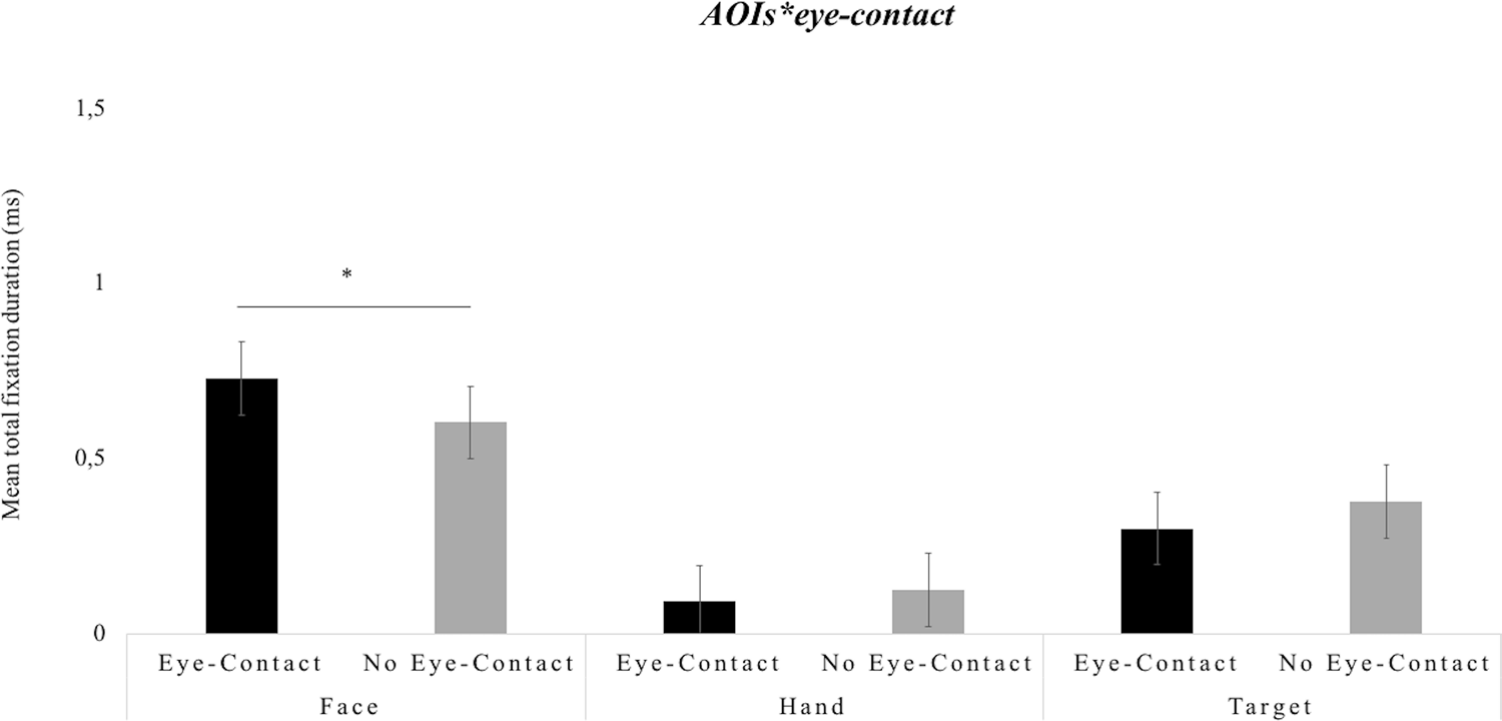
Infants’ total fixation duration scores. The graph shows the mean scores (ms) as a function of area of interest (Face, Hand, Target) and as a function of eye-contact (Eye-Contact, No Eye-Contact). The bars represent the standard error of the mean. * indicates significant differences.

Furthermore, the results showed various interaction effects. A significant interaction between AOI*eye-contact, *F*(2, 56) = 5.228, *p* < 0.01, *partial-η*^*2*^ = 0.15, *δ* = 0.81, revealed that the face was looked longer in the presence of eye-contact compared to the absence of eye-contact, *M*diff = 0.125, *SE* = 0.40, *p* < 0.01. Conversely, total fixation on the hand and target was not affected by the presence or absence of the initial eye-contact. This interaction is plotted in Fig. 2.

**Figure 3:**
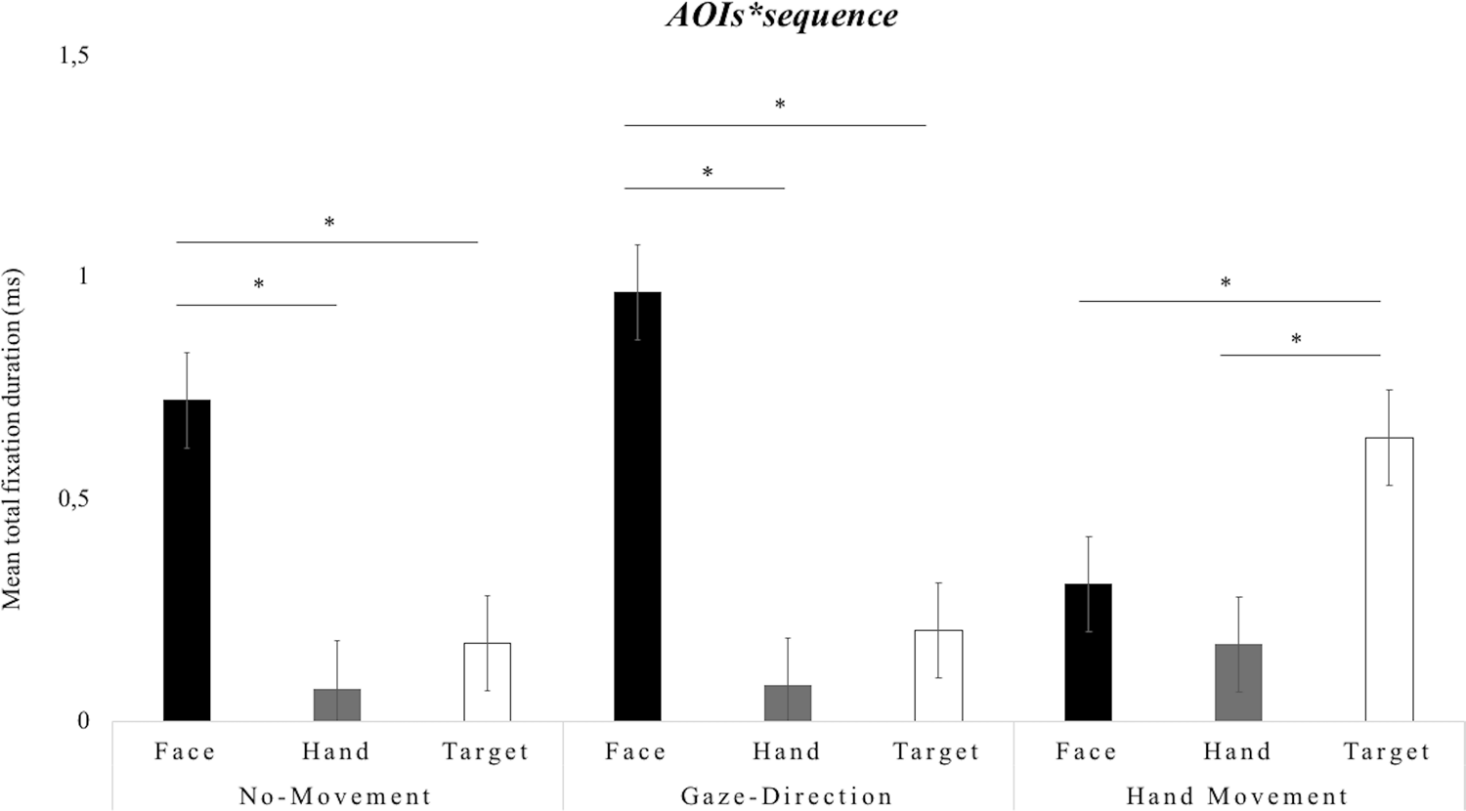
Infants’ total fixation duration scores. The graph shows the mean scores (ms) as a function of gaze (Congruent, Incongruent) and sequence (No-Movement, Gaze-Direction, Hand-Movement). The bars represent the standard error of the mean. * indicates significant differences.

Additionally, a significant two-way interaction AOI*sequence, *F*(4, 112) = 48.396, *p* < 0.001, *partial-η*^*2*^ = 0.15, *δ* = 0.81, revealed that the face was looked at longer in sequence 2 (Gaze-Direction) compared to both sequence 1 (No-Movement), *M*diff = 0.243, *SE* = 0.078, *p* < 0.05, and sequence 3 (Hand-Movement), *M*diff = 0.658, *SE* = 0.078, *p* < 0.001. Furthermore, the post-hoc analyses revealed that the hand was looked at longer in sequence 3 (Hand-Movement) compared to both sequence 1 (No-Movement), *M*diff = 0.100, *SE* = 0.028, *p* < 0.01, and sequence 2 (Gaze-Direction), *M*diff = 0.093, *SE* = 0.029, *p* < 0.05. Finally, the post-hoc analyses revealed that the target was looked at longer in sequence 3 (Hand-Movement) compared to both sequence 1 (No Initial Movement), *M*diff = 0.463, *SE* = 0.08, *p* < 0.001, and sequence 2 (Hand-Movement), *M*diff = 0.435, *SE* = 0.052, *p* < 0.001. This interaction is plotted in Fig. 3.

Finally, a significant three-way interaction (Table 1) gaze-direction*sequence*agency, *F*(2, 24) = 3.241, *p* < 0.05, *partial-η*^*2*^ = 0.104, *δ* = 0.595, revealed, for the human condition only, greater total fixation duration on all AOIs in sequence 2 (Gaze-Direction) compared to sequence 1 (No-Movement), for both gaze conditions (Congruence, Incongruence).

**Table 1:** Statistics comparing total fixation duration between sequences (1. No-Movement; 2. Gaze-Direction; 3. Hand-Movement) as function of agency (Human, Robot), gaze (Congruent, Incongruent).

Sequence	Gaze congruence			Gaze incongruence		
	Mean difference	Standard error	Sign	Mean difference	Standard error	Sign
**Human**						
1 versus 2	− **0.144**	0.043	**0.007**	− **0.101**	0.034	**0.018**
1 versus 3	0	0.068	1	− 0.047	0.041	0.794
2 versus 3	0.144	0.06	0.069	0.054	0.033	0.354
**Robot**						
1 versus 2	− 0.052	0.046	0.796	− 0.076	0.036	0.139
1 versus 3	− 0.121	0.073	0.326	− 0.031	0.044	1
2 versus 3	− 0.069	0.064	0.88	0.044	0.036	0.674

### Time to first fixation on the target

**Figure 4:**
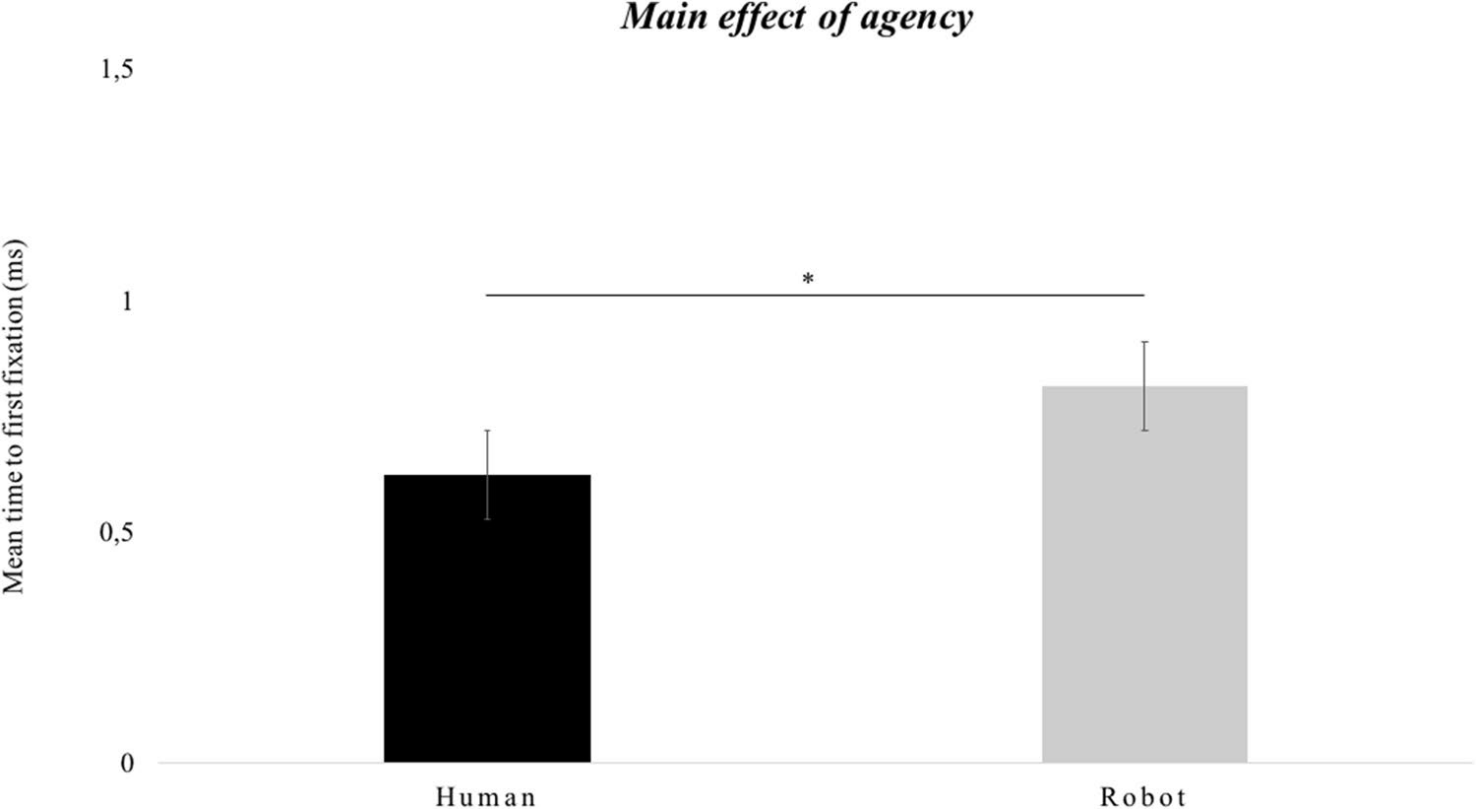
Infants’ time to first fixation. The graph shows the mean scores (ms) as a function of agency (Human, Robot). The bars represent the standard error of the mean. * indicates significant differences.

The results showed a main effect of *agency*, *F*(1, 28) = 4.891, *p* < 0.05, *partial-η*^*2*^ = 0.149, *δ* = 0.570, indicating that, independent of eye-contact and gaze direction, infants exhibited faster attendance to the target in the human condition compared to robot condition, *M*diff = − 0.192, *SE* = 0.087, *p* < 0.05 (Fig. 4).

**Figure 5:**
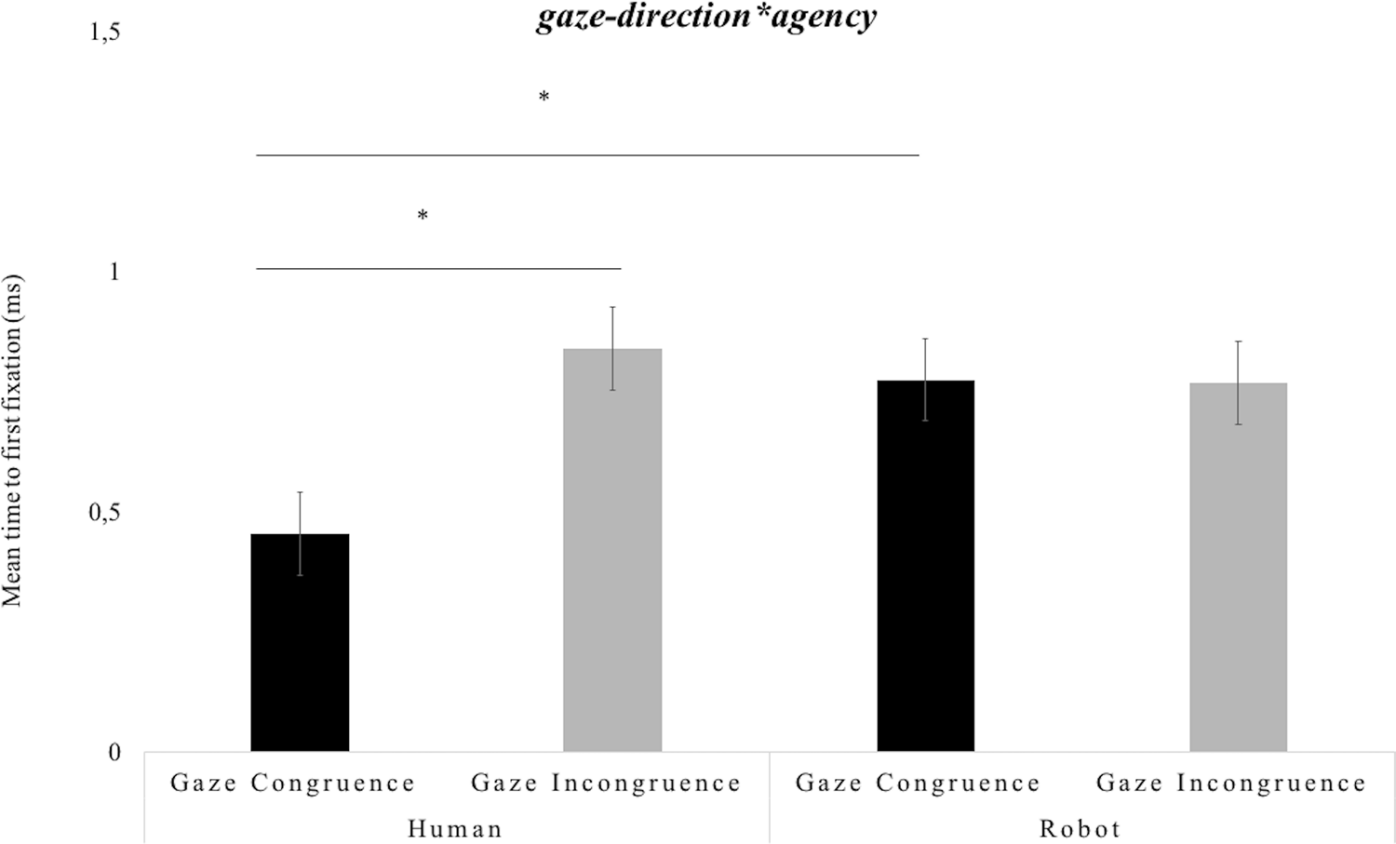
Infants’ time to first fixation scores. The graph shows the mean scores (ms) as a function of agent (Human, Robot) and gaze direction (Congruent, Incongruent). The bars represent the standard error of the mean. * indicates significant differences.

Additionally, the results showed a significant interaction between gaze-direction*agency, *F*(2, 27) = 5.516, *p* < 0.01, *partial-η*^*2*^ = 0.29, *δ* = 0.81. The post-hoc analyses revealed that infants’ attended faster to the target in the human gaze-congruent condition compared to human gaze-incongruent condition, *M*diff = -0.411, *SE* = 0.138, *p* < 0.005. This result was not found in the robot condition. Finally, infants’ time to first fixation on the target was faster in the human gaze-congruent condition compared to robot gaze-congruent condition, *M*diff = − 0.535, *SE* = 0.148, *p* < 0.001 (Fig. 5).

## Discussion

This study analysed 17-month-old infants’ sensitivity to human and robot’s referential gaze. To this purpose, the infants’ gazing behaviour was assessed while watching videos showing either a human or a humanoid robot (Robovie) performing an action anticipated by either congruent or incongruent gaze toward a target. The results on total fixation duration showed that infants paid the greatest attention to the face area of both agents, and were equally engaged by both agents’ initial eye-contact. However, infants showed to be particularly sensitive to the human gaze direction, as indexed by greater attention to head movement (compared to still face) in the human condition only. Sensitivity to the human gaze is also supported by data on time to first fixation, evaluating infants’ response to the agents’ referential gaze. The results showed that infants attended to the target faster when the action was performed by the human compared to the robot, and particularly in the presence of a congruent referential gaze.

With respect to the infants’ attention pattern to the video stimuli, indexed by total fixation time on specific AOIs (face, hand, object), the results showed that, independent of agent, infants preferred the face over both the hand and the target. Preference to the face was greater in the condition in which the action was primed by the agents’ direct eye-contact toward the infant. These findings are in line with previous data on humans showing that the face attracts greater attention compared to other body parts already from 6 months of age^36,37^. This effect is enhanced by the presence of direct eye-contact^37,38^. Interestingly, our data enrich previous findings suggesting that, besides the face^22^, also direct eye-contact of the robot exerts a similar attraction on the infant’s attention as that observed for the human. These results suggest that the robot’s anthropomorphic features and behaviours, such as the face and eye-contact, are able to activate similar attentional responses in infants aged 17 months. This might enable the robot to be ultimately perceived as a potential interactive partner. Nevertheless, we did find also differences in how the infant regards the referential nature of gaze between human and robot. First, our results on total fixation duration showed that infants paid greater attention to the human face in the sequence representing the gaze shift compared to the sequence in which there was not gaze shift. This effect was not found for the robot, suggesting that human referential gaze is more valuable (or informative) than the robot’s gaze. Additionally, data on time to first fixation on the target showed both faster shift to the target in the human gaze, with respect to the robot gaze, congruent condition, and faster response to the target in the congruent vs. incongruent condition, for the human and not the robot. Infants’ faster response to the congruent vs. incongruent human gaze shifts is in line with previous findings^17^, also suggesting that this effect emerges as early as at nine months^17^. As a counterproof, the differences between human and robot disappeared with gaze incongruence. These findings suggest that 17-months infants do not regard the robot's gaze as a social signal as they do for the human, where the inconsistency of gaze leads to a delayed response to the referential cue. Previous studies with 12 months infants^22^ have shown that, despite gaze following was similar when attending to the human or robot’s gaze, the human gaze was more informative and induced greater object learning compared to the robot’s gaze. This suggests that infants may be keen to treat humans as prevalent sources of information. Also, and congruently with our findings, 12-month-olds, but not 10-months old infants, showed greater anticipation time on the target object when primed by the human than the robot’s gaze^22^, thus supporting that infants privilege the referential nature of the human gaze compared to the robot’s gaze.

The mechanism of referential gaze recognition at 17 months is strictly related to human social cognition. The preferential attitude exhibited by infants for the referential gaze of a conspecifics compared to the gaze of a robot leads to at least two possible interpretations. The first one accounts for the specificity of a socio-cognitive mechanism, which would not allow the generalisation to other species or entities (e.g., robots). This would be in line with the hypothesis of a human species-specific eye-gaze mechanism suggested by studies demonstrating that infants prefer human gaze compared to the gaze of other living species, like monkeys^39^, or non-living ones, like robots^22,25^. The second interpretation would take on the experiential account and argue that preference for the human gaze is due to the inexperience that infants have with the gaze of other species or entities, like robots. Several studies have shown that gaze following develops within 12 months^33,40–45^, although the debate is still open on the exact onset period^5,7,9,24,31,46,47^. At this age, infants appear to be selectively attuned to follow the human gaze, and this would be in line with findings showing that 12-month old infants have difficulties in following the gaze of other species^39^. This selectivity may be plausibly due to extensive exposure to human interaction in early life. Generalization of gaze-following may then occur in subsequent stages of development, possibly through experience, i.e. learning. The importance of learning is supported by evidence suggesting that the mechanism of gaze-following gradually emerges through social experience^48–50^. Accordingly, infants would display preference for the human referential gaze because had not had the experience of the robot's gaze. The hypothesis of a narrowing of such inborn gaze sensitivity through extensive experience with human gaze is also supported by studies with precocial bird species suggesting that the mechanism for gaze following is highly conserved among vertebrates, is active at birth, and therefore it is not species specific^51,52^. Future studies could specifically address this interpretation by, for example, exposing infants of different ages to several sessions with robots and observe if sensitivity to referential gaze increases with experience. Also, robots could be placed in ecological settings for infants where they typically experience human interaction.

In sum, our data indicate, on the one hand, that children recognise the physical traits and behaviours that are salient for social interaction, such as the face and direct eye-contact, in both agents. Conversely, when agents perform an action—look at an object—, infants appear to be more sensitive to the human than the robot agency. This data leads to an important consideration regarding the possible presence of two distinct levels of gaze-following mechanism: a first preparatory level that focuses the infant's attention on the parts considered salient for interaction, allowing to recognise the other as a possible interactive partner; and a second level that evaluates whether the partner has agency. The results of the present study seem to suggest that at 17 months the first level of the gaze-following mechanism—recognise the other as a plausible interactional partner—is both active and generalised, while the second level—attribution of agency to the partner—is active with human but not (yet) generalised to other species or entities, i.e. robots.

## Conclusions

Infants at 17 months of age do not recognize the referential nature of the robot's gaze: this type of attribution, referential, is more readily made with conspecifics. Nevertheless, infants seem to be sensitive to some of the robot’s physical and behavioural human attributes, and namely its face and eye-contact. More specifically, data showed that children recognize, on the one hand, the robot as a plausible interactive partner but, at the same time, do not ascribe agency to it. These results allowed us to hypothesize two distinct processing levels of the gaze-following mechanism: the first one that evaluates if an entity is an interactive partner; the second one that evaluates if it is endowed with agency. Infants at 17 months of age seem to be particularly sensitive to human features also when embedded in a robot (first processing level), but they are not equipped with a generalization mechanism that enables them to regard the robot as a human-like agent (second processing level). As suggested, this mechanism may develop with experience.

## Limitations and future directions

Since at this age infants do not seem to react to the referential nature of the robot's gaze, it might be important to study whether these effects change as function of age. Additionally, it is important to underline that in our study the infants might have followed the head movement rather than just the eye-gaze. This choice was necessary because it was not possible to recreate in the robot the contrast between the dark pupil and the white sclera that guides eye-gaze perception. Therefore, future studies should find the way to test these two effects (head movement and eye-gaze) independently. Also, the results presented in this study were acquired with only one type of robot, while it might be interesting and important to evaluate the effect of robots differing in physical features, with different cues of embodiment/anthropomorphization. Moreover, it would be interesting to specifically compare in future related studies two alternative interpretations of the data: the idea of a species-specific gaze-following mechanism that is not generalized to robots vs. the lack of experience with robots. Finally, in line with current theoretical claims that studies with robots can be used to understand the nature of human psychological mechanisms^53,54^, this line of research with infants and robots can shed light on the mechanisms underpinning the development of early social cognition.

## Methods

### Participants

Data were obtained from an initial sample of 36 infants. Four infants were excluded from the analyses because of inattentiveness (one, whose gaze pattern was fuzzy; two who completed fewer than 3 trials of gazing; one for technical acquisition errors). The final sample was composed of thirty-two 17-month-old Japanese infants (F = 15; M = 17.43 months, SD = 0.96). The infants were divided into two groups for each condition as follows: (1) 16 infants for the Human condition (F = 8; M = 17.25 months, SD = 0.83); (2) 16 infants for the Robot condition (F = 7; M = 17.64 months, SD = 1.08). The children’s parents received a written explanation of the procedure of the study, the measurement items, and provided written informed consent before their infants took part in the study. The study was conducted in accordance to the Declaration of Helsinki and the experimental protocol was approved by the Research Ethics Review Board, Department of Psychology, Kyoto University, Japan.

### Design and stimuli

**Figure 6:**
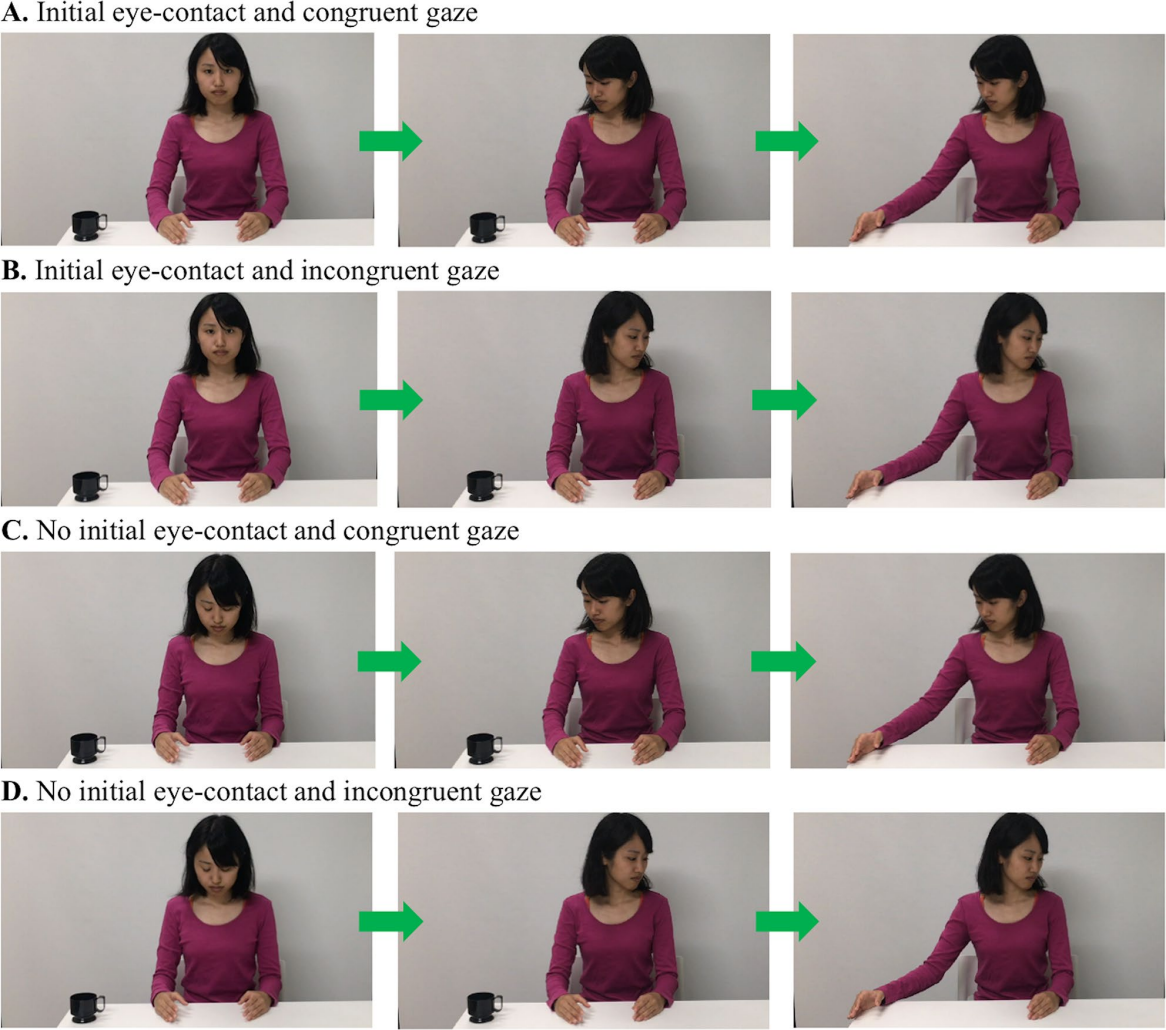
(**A**–**D**) Images representing the four human conditions (informed consent was obtained from the actress for publication of the images).

**Figure 7:**
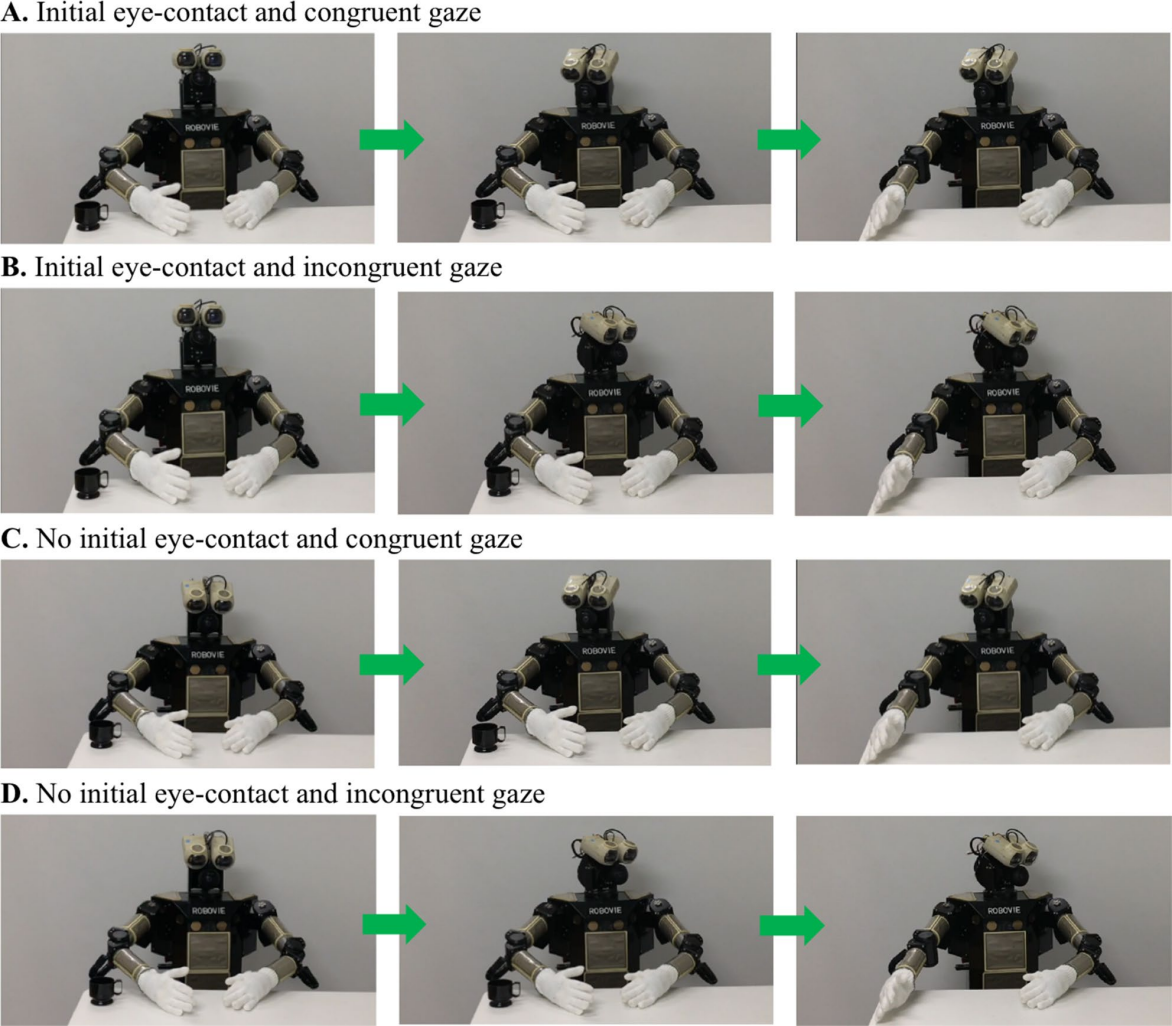
(**A**–**D**) Images representing the four robot conditions.

The design of the study was a multifactorial 2 × 2 × 3 × 3 × 2 mixed-model, with two levels of initial eye-contact (present, absent), two levels of gaze-congruence with the action (congruent, incongruent), 3 levels of area of interests (AOIs: face, hand, object), 3 levels of video sequence (no movement, head movement, hand movement) as the within-subject factors, and two levels of agency (Human, Robot) as the between-subject factor. The experimental stimuli where 4 video clips of the total duration of 6 s, in which either a human (a female; Fig. 6A–D) or the robot (Fig. 7A–D) gazed at a cup and then moved her/its right arm dropping the cup off the table. The size of the video stimuli is 1,280 × 1,024 pixels, while the AOIs were created of an equal size of 250 × 250 pixels.

Each video began with a scene in which the agent looked either at the camera or down at the table (2 s), thus defining the presence or absence of an initial eye-contact. Next, the agent turned toward and fixated on (2 s) the cup (congruent condition) or toward the portion of the table without the object (incongruent condition). In both conditions, the agent then moved her/its right arm (2 s) dropping the cup off the table. The human model maintained a neutral facial expression and remained silent throughout the entire sequence. Before each trial, an object (toy animation) appeared at the centre of the screen accompanied by a tinkling sound (2 s) to attract the infant’s attention.

Before the test videos, infants were administered a familiarization trial that displayed the human or robot upper body (5 s) followed by the presentation of a fixation point paired with a beeping sound (1 s). The aim of this trial was to familiarize the infants with the setting and the agents (human or robot).

### Procedure and apparatus

The infants were assessed individually in the presence of their mother in the Baby Centre at Kyoto University. Caregivers were instructed to close their eyes and to not talk to or interact with the infants during the task. The infants were randomly assigned to the Human or Robot condition and the presentation order of each condition (presence/absence of initial eye-contact; congruent/incongruent gaze direction) was randomized across infants.

We used the Tobi T60 (Tobii pro studio, Tobii Technology, Stockholm) to record the infant’s eye-gaze. The robot used for the tasks was the humanoid robot Robovie2 (Hiroshi Ishiguro Laboratories). The sampling rate of eye tracking was 60 Hz. The participants were seated on the caregiver’s lap approximately 60 cm from the monitor. Prior to recording, a five-point calibration was conducted. A clearview fixation filter was used for the eye-tracking data. Fixation was defined as gaze recorded within a 50-pixel diameter for a minimum of 200 ms, and this criterion was applied to the raw eye-tracking data to determine the duration of any fixation.

### Data analysis

The infants had to complete at least 3 out of 4 trials (video stimuli) to be included in the final analysis. More specifically, if more than one trial produced no data on the dependent variables (due to the infant’s disengagement or technical issues), the infant’s data were completely removed from the analysis. For each video, 3 AOIs were defined as follows: (1) the agent’s face, (2) the agent’s hand and (3) the target object (the cup). Face and hand have been defined as areas of interest in line with recent studies that demonstrate their importance as early social cues^35^. In addition, each video was divided into three sequences: (1) an initial sequence in which there was no movement or action by the protagonist; (2) a sequence that included the movement of the gaze towards the object or its opposite position; (3) a final sequence representing the completion of the action, i.e. dropping the object from the table.

The dependent variables were the following: (1) the total fixation duration to evaluate infants’ general attentional pattern on the different AOIs in the 3 sequences as a function of agency; (2) time to first fixation on the target in the final sequence showing the agent’s action completion (anticipation), and assessing infants’ sensitivity to the agents’ referential gaze. Time to first fixation was calculated as the time interval between the beginning of sequence 3 (hand movement towards the target object) and the onset of the saccade from the infant’s point of fixation at the end of the sequence 2 to the lateral target (in milliseconds). As outlined in the results, in sequence 2 infants fixated on the face area substantially more than on the target or the hand. Any possible differences in time to first fixation on the target in sequence 3 due to infants’ final gaze positioning in sequence 2 were counterbalanced by randomisation of conditions (initial eye contact/no eye contact, gaze congruence/gaze incongruence).

To evaluate infants’ general attentional pattern to the video stimuli, total fixation duration was entered in a repeated measures GLM analysis with 2 levels of *eye-contact* (Present, Absent), 2 levels of *gaze-direction* (Congruent, Incongruent), 3 levels of *sequence* (No-Movement, Gaze-Direction, Hand-Movement) and 3 levels of *AOIs* (Face, Hand, Target) as within-subjects factors, and 2 levels of *agency* (Human, Robot) as the between-subjects factor. To assess the effect infants’ sensitivity to the agents’ referential gaze, time to first fixation on the target was entered in a repeated measures GLM analysis carried out only on sequence 3, with 2 levels of *eye-contact* (Present, Absent), 2 levels of *gaze-direction* (Congruent, Incongruent) as within-subjects factors, and 2 levels of *agency* (Human, Robot) as the between-subjects factor. The Greenhouse–Geisser correction was used for violations of Mauchly’s Test of Sphericity (*p* < 0.05). Post-hoc comparisons were Bonferroni corrected.
