# Publisher Correction: The understanding of congruent and incongruent referential gaze in 17-month-old infants: an eye-tracking study comparing human and robot

**DOI:** 10.1038/s41598-020-77478-0

**Published:** 2020-12-10

**Authors:** F. Manzi, M. Ishikawa, C. Di Dio, S. Itakura, T. Kanda, H. Ishiguro, D. Massaro, A. Marchetti

**Affiliations:** 1 Research Unit on Theory of Mind, Department of Psychology, Università Cattolica del Sacro Cuore, Milan, Italy; 2 School of Graduated Letter, Department of Psychology, Kyoto University, Kyoto, Japan; 3 Centre for Baby Science, Doshisha University, Kyoto, Japan; 4 Human-Robot Interaction Laboratory, Department of Computer Science, Kyoto University, Kyoto, Japan; 5 Advanced Telecommunications Research Institute International, IRC/HIL, Keihanna Science City, Kyoto, Japan; 6 Department of Systems Innovation, Osaka University, Toyonaka, Japan

Correction to: *Scientific Reports* https://doi.org/10.1038/s41598-020-69140-6, published online 17 July 2020

This Article contains errors in Reference 20 which is incorrectly given as:

Cinzia, D. D. *et al.* Come i bambini pensano alla mente del robot. Il ruolo dell’attaccamento e della Teoria della Mente nell’attribuzione di stati mentali ad un agente robotico. *Sistemi intelligenti* **32**, 41–56. https://doi.org/10.1422/96279 (2020).

The correct Reference 20 appears below:

Di Dio, C. *et al*. Come i bambini pensano alla mente del robot. Il ruolo dell’attaccamento e della Teoria della Mente nell’attribuzione di stati mentali ad un agente robotico. *Sistemi intelligenti* **32**, 41–56. https://doi.org/10.1422/96279 (2020).

